# Identification of evolutionarily conserved downstream core promoter elements required for the transcriptional regulation of Fushi tarazu target genes

**DOI:** 10.1371/journal.pone.0215695

**Published:** 2019-04-18

**Authors:** Hila Shir-Shapira, Anna Sloutskin, Orit Adato, Avital Ovadia-Shochat, Diana Ideses, Yonathan Zehavi, George Kassavetis, James T. Kadonaga, Ron Unger, Tamar Juven-Gershon

**Affiliations:** 1 The Mina and Everard Goodman Faculty of Life Sciences, Bar-Ilan University, Ramat Gan, Israel; 2 Section of Molecular Biology, University of California, San Diego, La Jolla, CA, United States of America; Institute of Genetics and Molecular and Cellular Biology, FRANCE

## Abstract

The regulation of transcription initiation is critical for developmental and cellular processes. RNA polymerase II (Pol II) is recruited by the basal transcription machinery to the core promoter where Pol II initiates transcription. The core promoter encompasses the region from -40 to +40 bp relative to the +1 transcription start site (TSS). Core promoters may contain one or more core promoter motifs that confer specific properties to the core promoter, such as the TATA box, initiator (Inr) and motifs that are located downstream of the TSS, namely, motif 10 element (MTE), the downstream core promoter element (DPE) and the Bridge, a bipartite core promoter element. We had previously shown that Caudal, an enhancer-binding homeodomain transcription factor and a key regulator of the Hox gene network, is a DPE-specific activator. Interestingly, pair-rule proteins have been implicated in enhancer-promoter communication at the *engrailed* locus. Fushi tarazu (Ftz) is an enhancer-binding homeodomain transcription factor encoded by the *ftz* pair-rule gene. Ftz works in concert with its co-factor, Ftz-F1, to activate transcription. Here, we examined whether Ftz and Ftz-F1 activate transcription with a preference for a specific core promoter motif. Our analysis revealed that similarly to Caudal, Ftz and Ftz-F1 activate the promoter containing a TATA box mutation to significantly higher levels than the promoter containing a DPE mutation, thus demonstrating a preference for the DPE motif. We further discovered that Ftz target genes are enriched for a combination of functional downstream core promoter elements that are conserved among *Drosophila* species. Thus, the unique combination (Inr, Bridge and DPE) of functional downstream core promoter elements within Ftz target genes highlights the complexity of transcriptional regulation via the core promoter in the transcription of different developmental gene regulatory networks.

## Introduction

Gene regulatory networks governing developmental processes are tightly regulated at the transcription level [1–5]. Initiation of transcription of protein coding genes, miRNAs and long non-coding RNAs occurs at the Pol II core promoter, which encompasses the region from -40 to +40 bp relative to the +1 transcription start site (TSS). It is now established that the core promoter composition is a key component in the regulation of transcription (reviewed in: [6–13]). Core promoters may contain core promoter elements/motifs that confer specific properties to the core promoter, such as the TATA box [14], initiator (Inr) [15], and motifs that are located downstream of the TSS, namely, motif 10 element (MTE) [16, 17], the downstream core promoter element (DPE) [18–20] and the Bridge, a bipartite core promoter element (BridgeI and BridgeII) [21].

We had previously shown that the DPE motif has an important role in the gene network that controls the development of the anterior-posterior axis of the *Drosophila melanogaster* embryo [22]. Nearly all of the Hox genes contain DPE-dependent, TATA-less promoters. Moreover, we have shown that Caudal, a homeodomain sequence specific transcription factor, which is a key regulator of the Hox gene network, is a DPE-specific activator [22]. Caudal is the first identified core promoter-specific activator, and it is likely that additional transcriptional factors have a preference for a specific core promoter element. The concept of core promoter-specific activators is also supported by studies that demonstrate enhancer-promoter specificity (see for example, [23–25](. Interestingly, pair-rule proteins have been implicated in enhancer-promoter communication in the *engrailed* locus [26].

*fushi tarazu* (*ftz*) is one of the best-characterized pair-rule genes that activates segment polarity genes. It is a homeodomain transcriptional activator that is expressed in stripes (even numbered segments) very early in anterior-posterior axis formation [27–30] (reviewed in [31]). The specificity of Ftz target site selection is achieved by its interaction with its obligatory co-factor Ftz-F1 [32]. Ftz-F1 is an orphan nuclear receptor that is conserved in bilaterians. Ftz interacts with Ftz-F1 [33, 34] and cooperatively they bind to DNA as a complex, synergistically activating more than a dozen target genes [32, 35–38]. Notably, the interaction between the Ftz protein itself and DNA is very weak [35], and the specificity of Ftz target site selection is achieved by Ftz-F1, whose DNA binding specificity is much more stringent than that of Ftz [32, 37, 39, 40].

We have previously demonstrated that the *ftz* promoter contains both a TATA-box and a DPE motif, and that Caudal preferentially activates the *ftz* promoter through the DPE motif [22]. Thus, we decided to examine whether the Ftz transcription factor, which shares sequence homology with Caudal, also has a preference for activating transcription of its target genes in a core-promoter-specific manner. In this study, we have discovered that Ftz and Ftz-F1 synergistically activate transcription with a preference for the DPE motif, and that Ftz target genes are enriched for a combination of functional downstream core promoter elements that are conserved among *Drosophila* species.

## Material and methods

### Expression plasmids

Ftz and Ftz-F1 coding regions were cloned into the pAc5.1 ⁄ V5-His C expression vector (Thermo Fisher Scientific) without any tags. To that end, RNA was purified from 0–12 hours old *Drosophila* embryos using the Trizol reagent (Thermo Fisher Scientific) and reverse transcribed into cDNA using MMLV-Reverse Transcriptase (Promega). The primers that were used for cloning Ftz (forward: 5’ AAGGTACCATGGCCACCACAAACAGC 3’, reverse: 5' AAGGATCCTCATCAAGACAGATGGTAGAGGTCC 3') include a KpnI restriction site in the forward primer and a BamHI restriction site in the reverse primer. The coding region of Ftz-F1 was amplified using the Berkeley *Drosophila* Genome Project (BDGP) LD15303 clone as a template. The primers that were used for cloning Ftz-F1 (Forward: 5’ CCGAATTCATGGATACCTTCAATGTACCTATGCTGGCGGAGAG 3’, Reverse: 5’ TTGGATCCTACTATCCCTTGCGCTTGGCGTGCAG 3’) include an EcoRI restriction site in the forward primer and a BamHI restriction site in the reverse primer. All PCR reactions were carried out using PfuUltra II polymerase (Agilent Technologies). PCR fragments were initially cloned into the pGEM-T Easy vector (Promega) before cloning into the final expression plasmids. The DNA sequence of each plasmid was verified by sequencing reactions (Hy Labs).

### Luciferase reporter plasmids

Each synthetic Ftz—Ftz-F1 firefly luciferase reporter plasmid contains a total of six Ftz DNA binding sites (CCACAATTAGG) and three Ftz-F1 DNA binding sites (TCCGAAGGACAC), based on the *engrailed* intronic enhancer [32]. The synthetic binding sites were cloned upstream of the *ftz* core promoter and the firefly luciferase reporter gene of a pGL3-Basic plasmid with a modified polylinker. The DNA binding sites were assembled using three sets of annealed top and bottom oligonucleotides, each containing a Ftz-F1 DNA binding site flanked by Ftz DNA binding sites from either side (Ftz binding sites are in bold, Ftz-F1 binding sites are underlined). The three sets were ligated to each other and to the vector using EcoRI, XhoI, PstI and SpeI compatible restriction enzyme sites (designated by lower case letters):

aattc**CCACAATTAGG**AATCCGAAGGACACTG**CCACAATTAGG**ctcgag**CCACAATTAGG**AATCCGAAGGACACTG**CCACAATTAGG**ctgcag**CCACAATTAGG**AATCCGAAGGACACTG**CCACAATTAGG**a. The synthetic binding sites were cloned into either a DPE-dependent or a TATA-dependent *ftz* promoter firefly luciferase reporter plasmid [22], which are identical except for the sequences at the DPE and TATA regions. For cloning the minimal promoters of Ftz and Ftz-F1 target genes into a reporter plasmid, double-stranded oligonucleotides comprising core promoter sequences from –10 to +40 were inserted into the EcoRI and PstI sites of a pGL3-Basic plasmid with a modified polylinker. A 7-nucleotides DPE mutation was generated by replacing the wild type (WT) sequence with CTCATGT in positions +28 to +34 relative to the A_+1_ of the Inr. Mutations in BridgeI were generated by replacing the WT sequence with ATCCA in position +18 to +22 relative to the A_+1_ of the Inr [21]. A partial DPE mutation was generated by replacing the WT sequence with GTA in position +27 to +29 relative to the A_+1_ of the Inr. Promoter sequence variants are listed in Table 1.

**Table 1:** Minimal promoter sequence variants of Ftz target genes examined in this study.

gene	variant	Analyzed promoter sequence (-10 to +40)
*en*	WT	gtagtcaaCTAATTCagtcgttgcgctCGATGtgaacAGACGTgcgtgtc
	mDPE	gtagtcaaCTAATTCagtcgttgcgctCGATGtgaac**CTCATGt**cgtgtc
	mBridgeI	gtagtcaaCTAATTCagtcgttgcgct**ATCCA**tgaacAGACGTgcgtgtc
	mDPE_27-29_	gtagtcaaCTAATTCagtcgttgcgctCGATGtgaa**gTA**ACGTgcgtgtc
*drm*	WT	gtaactttGCAGTTgactctcgcgcacAGAAGcgttcGGAAGTgaaatat
	mDPE	gtaactttGCAGTTgactctcgcgcacAGAAGcgttc**CTCATGt**aaatat
	mBridgeI	gtaactttGCAGTTgactctcgcgcac**ATCCA**cgttcGGAAGTgaaatat
	mDPE_27-29_	gtaactttGCAGTTgactctcgcgcacAGAAGcgtt**gTA**AAGTgaaatat
*Sema5C*	WT	tggtgggtTCAGTTtcttgcgactcttTGCGCggtcaACTTCGccgatcg
	mDPE	tggtgggtTCAGTTtcttgcgactcttTGCGCggtca**CTCATGt**cgatcg
	mBridgeI	tggtgggtTCAGTTtcttgcgactctt**ATCCA**ggtcaACTTCGccgatcg
	mDPE_27-29_	tggtgggtTCAGTTtcttgcgactcttTGCGCggtc**gTA**TTCGccgatcg
*Ppa*	WT	ctgcgagtTCAGTTtttctttatcatcCGGTTcgtgcACATCGcgtctcg
	mDPE	ctgcgagtTCAGTTtttctttatcatcCGGTTcgtgc**CTCATGt**gtctcg
	mBridgeI	ctgcgagtTCAGTTtttctttatcatc**ATCCA**cgtgcACATCGcgtctcg
	mDPE_27-29_	ctgcgagtTCAGTTtttctttatcatcCGGTTcgtg**gTA**ATCGcgtctcg
*Cad74A*	WT	aagtccggCCAGAAggtaagcgagcgtCGCTTcgtacAGACGTggtcgcg
	mDPE	aagtccggCCAGAAggtaagcgagcgtCGCTTcgtac**CTCATGt**gtcgcg
	mBridgeI	aagtccggCCAGAAggtaagcgagcgt**ATCCA**cgtacAGACGTggtcgcg
	mDPE_27-29_	aagtccggCCAGAAggtaagcgagcgtCGCTTcgta**gTA**ACGTggtcgcg
*noc*	WT	cgttgaatTCAATTcgaattttgacttCGCAGcgttcAGACGTgttcgga
	mDPE	cgttgaatTCAATTcgaattttgacttCGCAGcgttc**CTCATGt**ttcgga
	mBridgeI	cgttgaatTCAATTcgaattttgactt**ATCCA**cgttcAGACGTgttcgga
	mDPE_27-29_	cgttgaatTCAATTcgaattttgacttCGCAGcgtt**gTA**ACGTgttcgga
*opa*	WT	ggagcattTCAGTCctgctgcgcatctTGAAAcgtcaAGTCTTggcattc
	mDPE	ggagcattTCAGTCctgctgcgcatctTGAAAcgtca**CTCATGt**gcattc
	mBridgeI	ggagcattTCAGTCctgctgcgcatct**ATCCA**cgtcaAGTCTTggcattc
	mDPE_27-29_	ggagcattTCAGTCctgctgcgcatctTGAAAcgtc**gTA**TCTTggcattc

Nucleotides’ match to core promoter element position weight matrices (PWMs) of Inr, BridgeI and DPE are shown by uppercase letters. Mutated nucleotides are shown in bold. The PWMs used to detect the core promoter motifs are the same as described in [48].

The *Scr* (*Sex combs reduced*)-*Renilla* luciferase reporter was constructed by replacing the firefly luciferase gene in the *Scr*-pGL3 Basic, which contains a genomic *Scr* fragment encompassing from −3103 to +110 relative to A_+1_ in the Inr [22], with the coding region of the *Renilla* luciferase gene. The coding region of the *Renilla* luciferase gene was amplified by PCR with the following primers: forward 5’ GAAGATCTGCCACCATGACTTCGAAAGTTTATGATCC 3’ and reverse 5’ GCGGCCGCTCTAGAATTATTGTTC 3’ (restriction enzyme sites are underlined) using *Pol III*-*Renilla* luciferase reporter plasmid (kind gift of Norbert Perrimon, Harvard Medical School) as a template. The DNA sequence of each plasmid was verified by sequencing reactions (Hy Labs).

### Transient transfections and reporter gene assays

*Drosophila* Schneider S2R+ adherent cells were cultured in Schneider’s *Drosophila* Medium (Biological Industries) that was supplemented with 10% heat-inactivated fetal calf serum. Cells were transfected in 24-well plates by using the Escort IV reagent (Sigma-Aldrich). For dual luciferase assays, cells were plated at 6 x 10^5^ cells per each well of a 24-well plate one day prior to transfection. Each well was transfected with a total of 1 μg DNA composed of 930 ng of a vector control (mock -pAc empty), 60 ng of firefly luciferase Ftz and Ftz-F1 target gene reporter constructs and 2 ng of *Scr-Renilla* luciferase reporter. Co-activation experiments were performed by transfection of 25 ng of each expression vector (Ftz, Ftz-F1 or vector control). For the analyses of minimal core promoter constructs, no activator was added. Medium was replaced one day after transfection, and cells were harvested 36–48 hrs post transfection and assayed for dual luciferase activities, as specified by the manufacturer (Promega). To correct for variations in transfection efficiency, firefly luciferase activity of each sample was normalized to the corresponding *Renilla* luciferase activity. Each transfection was performed in triplicates, and each graph represents an average of at least 3 independent experiments.

### In vitro transcription and primer extension analysis

*In vitro* transcription reactions with *Drosophila* embryo nuclear extracts were carried out as previously described [41–43] using 400–500 ng of supercoiled DNA templates with *Drosophila* high-salt nuclear extracts [44]. The resulting transcripts were subjected to primer extension analysis with a radiolabeled reverse luciferase primer (5’ TCTTCCAGCGGATAGAATGGCGCC 3’). All experiments were carried out a minimum of three independent times to ensure reproducibility of the data. Image processing and quantification of reverse transcription products were carried out using either ImageJ or Image Gauge v.3.0 (Fuji Photo Film Co.).

### Calculation of core promoter element frequency

The list of Ftz target genes was composed of both previously characterized Ftz target genes [32, 33, 35, 36, 45, 46] and all target genes identified by ChIP-chip data (pooled from Ftz “gene expression profile analysis”, “ChIP-chip binding sites mapping” and “direct targets” lists) [47]. Core promoter element compositions were taken from the CORE database [48], containing all *Drosophila* transcripts based on CAGE data [49]. Only peaked or unclassified promoter types were analyzed, and different transcripts of the same gene were considered separately. Transcripts were assigned to mutually exclusive groups based on their core promoter composition. The proportion of each core promoter combination for Ftz target genes was compared to the expected one (based on the *Drosophila* genome) using the chi-square test. P-values were adjusted using Bonferroni correction for multiple testing.

### phyloP conservation analysis of the downstream core promoter region

The TSSs positions of each *Drosophila melanogaster* transcript were downloaded from the EPDnew database (http://epd.vital-it.ch) [50, 51]. The exon coordinates of every *Drosophila melanogaster* transcript were downloaded from the UCSC table browser (https://genome.ucsc.edu/cgi-bin/hgTables) “RefSeq All (ncbiRefSeq)” table, which is part of the “NCBI RefSeq” track (within the “Genes and Gene Predictions” group).

The sequence conservation of the -2 to +33 promoter regions of the *en*, *drm*, *Sema5c*, *Ppa*, *Cad74A*, *noc* and *opa Drosophila melanogaster* genes and of the corresponding exons was analyzed using the UCSC “PhyloP (phyloP27way)” table, which is part of the “Conservation” track (within the “Comparative Genomics” group). The phyloP utility is part of the PHAST (Phylogenetic Analysis with Space/Time models) package for comparative and evolutionary genomics [52, 53]. The average exon conservation of a 35 bp window was calculated by locating windows of 35 bp, starting from a random position within the gene exons. This process was repeated a 1000 times. The distribution of the average conservation scores of randomly chosen 35 bp sequences within gene exons was plotted and compared to the average phyloP conservation scores of the 35 bp located at -2 to +33 relative to the TSS of the specific *Drosophila melanogaster* gene. If the average conservation score of the motif sequence falls in the boundaries ±2 STD in the distribution graph, the null hypothesis is not refuted *i*.*e*., the conservation level of the area of motif sequence (downstream core promoter region) is similar to the conservation level of its related gene exons. A 35 bp sequence located 200 bp away from the core promoter region (from -241 to -275) was downloaded using the NCBI genome data viewer (https://www.ncbi.nlm.nih.gov/genome/gdv/) and used for comparison.

### Evolutionary conservation analysis of the Inr, BridgeI and DPE combination

Evolutionary conservation analysis of the Inr, BridgeI and DPE combination within the core promoters of seven Ftz target genes (*en*, *drm*, *Sema5c*, *Ppa*, *cad74A*, *noc* and *opa*) was performed using the dm6 *Drosophila melanogaster* genome assembly. As the TSSs of these *Drosophila melanogaster* genes were experimentally defined, the Multiz alignment from the UCSC genome browser (http://genome.ucsc.edu) of *Drosophila melanogaster* and 26 additional insect species, as well as 6 additional close gene homologs were used towards the analysis of sequence conservation, as detailed below. For every *Drosophila melanogaster* transcript, the position of the TSS was downloaded from EPDnew database (http://epd.vital-it.ch) [50, 51]. Based on this position, the UCSC 27 insects Multiz alignments of the genome sequence ±500 bp from the TSS were downloaded from UCSC genome browser “Multiz Align (multiz27way)” table, which is part of the “Conservation” track (within the “Comparative Genomics” group). The insects included in the Multiz alignment are: *Drosophila melanogaster*, *Drosophila simulans*, *Drosophila sechellia*, *Drosophila yakuba*, *Drosophila erecta*, *Drosophila biarmipes*, *Drosophila suzukii*, *Drosophila ananassae*, *Drosophila bipectinata*, *Drosophila eugracilis*, *Drosophila elegans*, *Drosophila kikkawai*, *Drosophila takahashii*, *Drosophila rhopaloa*, *Drosophila ficusphila*, *Drosophila pseudoobscura*, *Drosophila persimilis*, *Drosophila miranda*, *Drosophila willistoni*, *Drosophila virilis*, *Drosophila mojavensis*, *Drosophila albomicans*, *Drosophila grimshawi*, *Musca domestica*, *Anopheles gambiae*, *Apis mellifera and Tribolium castaneum*. A position weight matrix (PWM) that combines the three core promoter motifs (*Drosophila melanogaster* Inr, BridgeI and DPE) was constructed based on the individual PWMs of these motifs, used in the core promoter Elements Navigation Tool (ElemeNT) [48]. For *en* and *Cad74A*, the mammalian Inr (instead of the *Drosophila melanogaster* Inr) PWM was used, as these genes contain an Inr sequence that conforms to the mammalian Inr. For each of the sequences that were downloaded from Multiz, a program was run to detect the existence of putative elements whose PWM similarity is above a specified threshold. As listed above, most of the species’ genome sequences that were downloaded from the UCSC Multiz alignment belong to the *Drosophiladea* family. To check whether the combined motif also exists in gene homologs in insect families other than the *Drosophiladea* family, the *Drosophila melanogaster* proteins encoded by each of the analyzed transcripts were downloaded from NCBI (https://www.ncbi.nlm.nih.gov/). For each protein, protein blast (BLASTP) was run on non-redundant protein sequences (nr) database excluding the *Drosophilidae* family (taxon identifier 7214). From the list of results of each one of the BLASTP queries, the top six species with highest coverage & identity were chosen. The gene coordinates for each of these species were downloaded from NCBI. Since there was no available information on the transcription start sites in these species, the sequence of ±1000 bp around the annotated gene start position was downloaded from NCBI. Similarly to the process run for the sequences downloaded from UCSC Multiz alignment, using a PWM that combines the three core promoter motifs (Inr, BridgeI and DPE), a program was run to detect the existence of putative elements whose PWM similarity is above a specified threshold on every one of the downloaded sequences. Motif sequences with PWM scores higher than 5 that were located within ±200 bp from the TSS or the annotated gene start positions were chosen. With these defined thresholds, there was only one motif detected for most species. In case more than one motif was detected per species, the motif that was closer to the TSS or gene start was chosen. The motif logos were generated using WebLogo (https://weblogo.berkeley.edu/) [54, 55].

## Results

### Ftz and its co-factor Ftz-F1 preferentially activate promoters via the DPE motif

Caudal is a homeodomain sequence-specific DNA binding transcription factor that has been shown to bind and activate the transcription of *ftz* [56]. We had previously demonstrated that the core promoter of the *ftz* gene contains a TATA box, an Inr and a DPE motif [22]. Moreover, we had shown that Caudal, as well as the mouse Caudal-related Cdx proteins, activate transcription of the *ftz* promoter with a distinct preference for the DPE motif over the TATA box [22, 57]. We postulated that there are additional sequence-specific transcription factors that activate transcription with a preference for a specific core promoter composition. Ftz, another homeodomain protein and a direct target of Caudal, is a natural candidate. The similarity between Ftz and Caudal is further supported by BLASTP analysis, which indicates that Caudal and Ftz contain two similar sequence stretches (S1 Fig). Thus, we have decided to test whether Ftz could activate transcription with a preference for the DPE motif.

It has been shown that the specificity of Ftz activity is achieved by its interaction with its obligatory co-factor, the orphan nuclear receptor Ftz-F1 [32, 37, 58]. Ftz and Ftz-F1 form a stable complex and bind cooperatively to DNA, resulting in synergistic activation of multiple target genes [33, 36, 58]. We therefore sought to examine whether Ftz and Ftz-F1 activate their target genes with a preference for the DPE motif, as was previously reported for Caudal.

**Fig 1:**
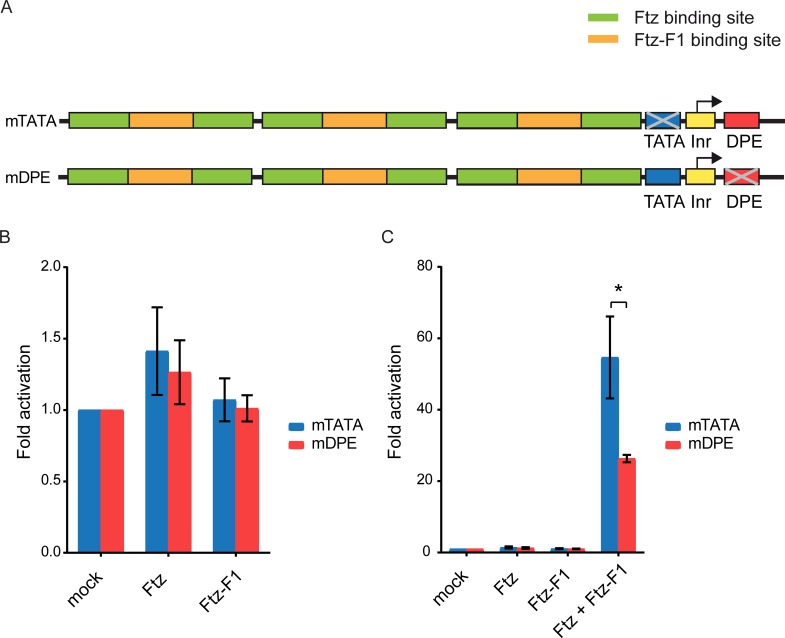
Transcriptional activation of synthetic reporter constructs by Ftz and Ftz-F1. (A) A schematic illustration of synthetic Ftz and Ftz-F1 reporter constructs. The synthetic binding sites were cloned upstream of the *ftz* core promoter and the firefly luciferase reporter gene. Each firefly luciferase reporter plasmid contains either a mutated TATA box (mTATA) or a mutated DPE motif (mDPE). The diagram is not drawn to scale. (B and C) *Drosophila* S2R+ cells were transfected with 25ng of each Ftz and Ftz-F1 expression vectors, as well as 60 ng of Ftz-Ftz-F1 reporter plasmids and 2 ng of *Scr-Renilla* luciferase reporter plasmid, and assayed for Dual-Luciferase activity. To correct for variations in transfection efficiency, the firefly luciferase activity of each sample was normalized to the corresponding *Renilla* luciferase activity. Graph represents an average of three independent experiments. Error bars represent the S.E.M. (B) Graph represents the fold activation values of the reporter plasmids by either Ftz or Ftz-F1 expression plasmid alone. (C) Graph represents the fold activation values of the reporter plasmids by Ftz, Ftz-F1 or Ftz and Ftz-F1 expression plasmids of the same data shown in (B). Note the different Y-axis scale compared to panel B. * p<0.05.

In order to test whether Ftz and Ftz-F1 activate their target genes with a preference for the DPE motif, we generated firefly luciferase reporter plasmids containing synthetic Ftz and Ftz-F1 binding sites derived from the *engrailed* enhancer [32], upstream of a *ftz* DPE-dependent core promoter (containing a mutation in the TATA-box; mTATA) or a *ftz* TATA-dependent core promoter (containing a mutation in the DPE; mDPE) (Fig 1A). Importantly, these synthetic reporter plasmids are identical except for the sequences at the DPE and TATA regions. *Drosophila* S2R+ cells, which do not express *ftz* RNA and only express low levels of *ftz-f1* RNA (modENCODE, [59]), were co-transfected with expression vectors for Ftz and/or Ftz-F1, as well as with a synthetic DPE- or TATA-dependent firefly luciferase reporter plasmid and a control *Renilla* luciferase plasmid. Cells were harvested 36–48 h post-transfection and assayed for dual-luciferase activity.

Neither Ftz nor the Ftz-F1 co-factor alone significantly activated the TATA-dependent (mDPE) or the DPE-dependent (mTATA) synthetic reporter (Fig 1B). The synergistic effect of Ftz and Ftz-F1 reported in the literature [32, 35–37] was observed, with the mTATA reporter being activated ~55 fold and the mDPE reporter activated ~26 fold (Fig 1C). Remarkably, co-transfected Ftz and Ftz-F1 activate the synthetic reporters with a preference for the DPE (Fig 1C). Thus, in addition to sharing homologous sequences with Caudal, this data suggests that Ftz and Ftz-F1 are also able to activate transcription to different levels depending on the core promoter composition.

### The downstream core promoter region of Ftz target genes is enriched for a combination of Inr, Bridge and DPE motifs

**Fig 2:**
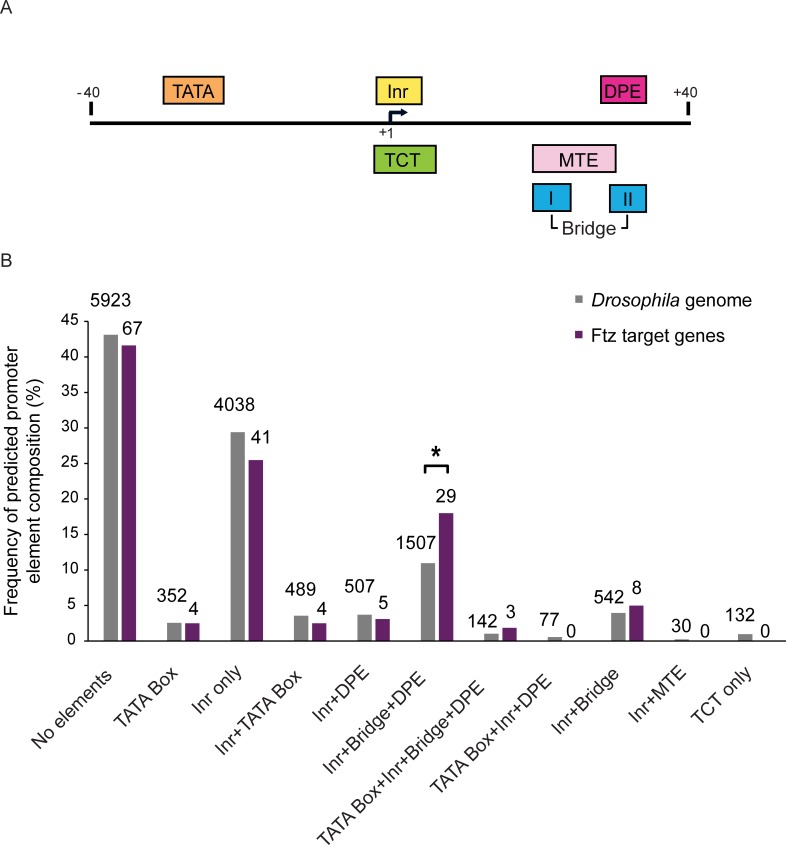
Frequency of predicted core promoter composition of the *Drosophila* genome, as compared to Ftz target genes. (A) A schematic illustration of the most common core promoter elements found in focused *Drosophila melanogaster* promoters. (B) *Drosophila* transcripts that initiate transcription from CAGE-defined regions [49] were annotated *in-silico* for core promoter composition, and the proportion of each core promoter composition of *ftz* target genes was compared to the expected one using the chi-square test. The absolute number of promoters in each category is denoted above each column. The total number of CAGE-defined transcripts was 13964 for the whole genome and 285 for Ftz targets. P-values were adjusted using Bonferroni correction for multiple testing. An asterisk denotes a p-value of 0.042.

To investigate whether Ftz target genes are regulated via a specific core promoter element, we first analyzed all Ftz targets based on the previously characterized target genes [32, 33, 35, 36, 45, 46] and on ChIP-chip analysis [47]. We used the *Drosophila melanogaster* CORE database and the Elements Navigation Tool (ElemeNT) [48], which annotates CAGE-defined *Drosophila melanogaster* TSSs [49] and analyzed the core promoter composition of these genes. Interestingly, many of the Ftz target genes contain downstream core promoter elements, such as the Bridge, DPE, and MTE. Remarkably, analysis of the fraction of genes containing a unique combination of Inr, DPE and Bridge core promoter configuration shows a statistically significant enrichment among Ftz target genes, as compared to the distribution of core promoter elements within the *Drosophila* genome (Fig 2). By definition, this combination of downstream core promoter elements is distinct from the Inr and MTE combination, which prompted us to investigate this unique combination.

### The Bridge and DPE motifs of multiple Ftz target genes are evolutionarily conserved

To assess the potential contribution of the downstream core promoter region to the regulation of Ftz target genes, we analyzed the sequence conservation of the -2 to +33 promoter regions of seven *Drosophila melanogaster* Ftz target genes. The analyzed genes included the well-characterized *en* [32, 35, 38], as well as the *drm*, *Sema5c*, and *noc* Ftz target genes identified in Ftz-F1 mutants [36]. In addition, the less characterized *Ppa*, *Cad74A* and *opa* genes [47, 60] were also tested. Each of these target genes contains a potential Inr, Bridge and DPE combination, with the exception of *opa*, which only contains an Inr and DPE. Interestingly, *en* and *Cad74A* sequences conform to the mammalian initiator sequence, while the other promoter sequences match both *Drosophila* and mammalian initiator motifs. All of the examined genes are co-expressed with Ftz and Ftz-F1 during early embryonic stages of development (modENCODE data in FlyBase [59], S2 Fig).

**Fig 3:**
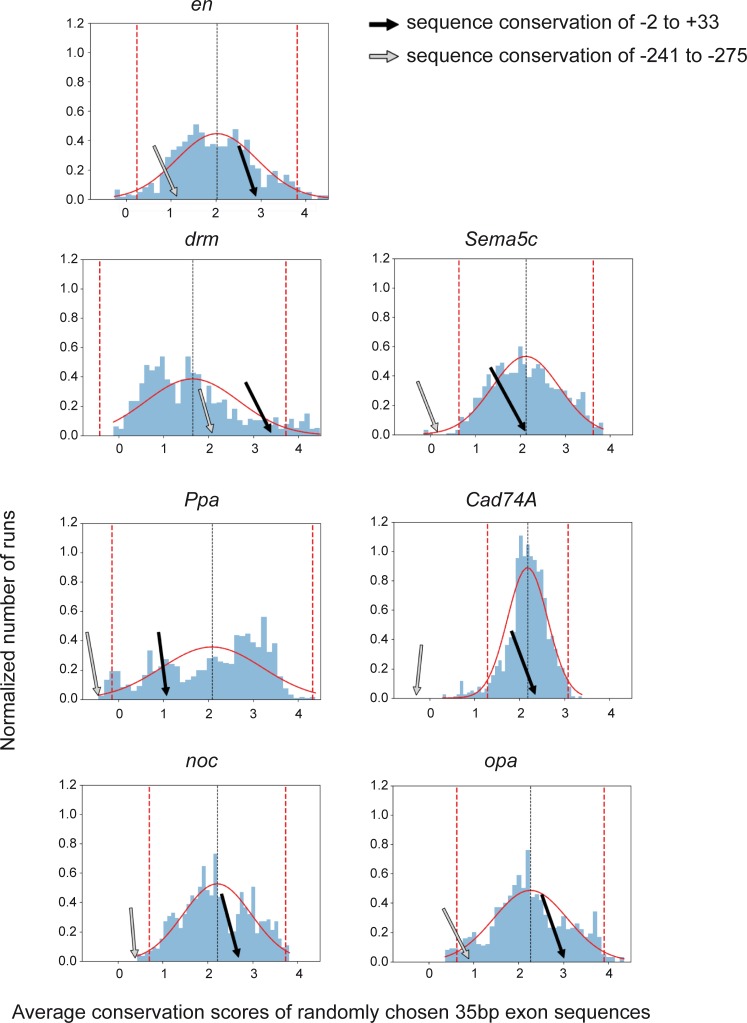
The conservation level of the downstream core promoter region is similar to the conservation level of its related gene exons. Sequence conservation analysis (using phyloP) was done to compare the conservation of the -2 to +33 promoter region (indicated by a black arrow) of seven characterized *Drosophila melanogaster* Ftz target genes to the average conservation scores of randomly chosen 35 bp sequences within its related gene exons. The conservation of a 35 bp sequence located 200 bp away from the core promoter region (from -241 to -275) is indicated by a gray arrow.

We first calculated the distribution of the average conservation scores of 1000 randomly chosen 35 bp sequences within exons of each of the abovementioned genes. Since the coding region is known to be highly conserved, the calculated conservation scores were used as a reference for the conservation level of the -2 to +33 region of the corresponding gene. The conservation analysis was based on the phyloP utility within the PHAST (Phylogenetic Analysis with Space/Time models) package for comparative and evolutionary genomics [52, 53]. The analysis demonstrated that the -2 to +33 region of the promoter of each of the seven target genes is within the boundaries of ±2 STD from the average conservation scores of randomly chosen 35 bp sequences within the exons of that gene (Fig 3), indicating that the evolutionary conservation level of the downstream core promoter region of each tested target gene is similar to the conservation level of its related exons.

We then used a 35 bp sequence located 200 bp upstream of the core promoter region (from -241 to -275) as a control. This upstream sequence was conserved for *en*, *drm* and *opa*, but not for *Sema5c*, *Ppa*, *Cad74A* and *noc*. Notably, the conservation levels of the core promoter regions of all analyzed Ftz target genes were higher than the conservation levels of the upstream control regions, further implicating the conserved downstream core promoter region in the regulation of these genes.

**Fig 4:**
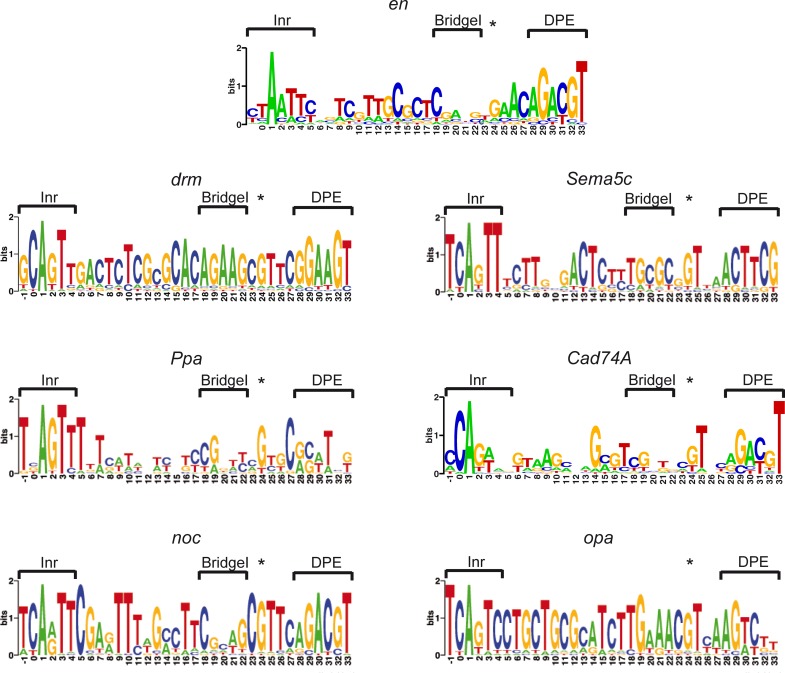
The core promoters of multiple Ftz target genes contain conserved Inr, BridgeI and DPE elements. Motif logos for evolutionarily conserved Inr, BridgeI and DPE combinations were generated using WebLogo (weblogo.berkeley.edu). The species that contributed to the logos are listed in Table 2.

We next analyzed the sequence conservation of the -2 to +33 nucleotides within the core promoters of each of these seven *Drosophila melanogaster* Ftz target genes using the Multiz alignment from the UCSC genome browser (http://genome.ucsc.edu), which includes *Drosophila melanogaster*, 26 additional insect species, and 6 additional close gene homologs (Table 2). The generated motif logos for the -2 to +33 promoter regions of *en*, *drm*, *Sema5c*, *Ppa*, *Cad74A* and *noc*, demonstrate that the combination of Inr, Bridge and DPE and its strict spacing requirement are evolutionarily conserved in multiple *Drosophila* species, as well as non-*Drosophila* insects (Fig 4 and Table 2). The *opa* promoter, which does not contain a BridgeI sequence motif, is also conserved. Interestingly, a G nucleotide at position +24 was previously identified as overrepresented in Inr-DPE containing promoters [20]. Indeed, the motif logos of all seven promoters contain a G nucleotide at this position, between the BridgeI and DPE motifs, highlighting the existence of additional evolutionary conserved positions within the downstream core promoter region.

**Table 2:** The -2 to +33 nucleotides within the core promoters of seven *Drosophila melanogaster* Ftz target genes are conserved to multiple insect species.

Gene Name /	*en*	*drm*	*Sema5c*	*Ppa*	*Cad74A*	*noc*	*opa*
Species name							
*Drosophila melanogaster*	+	+	+	+	+	+	+
*Drosophila simulans*	+	+	+	-	+	+	+
*Drosophila sechellia*	+	+	+	+	+	+	+
*Drosophila yakuba*	+	+	+	+	+	+	+
*Drosophila erecta*	+	+	+	+	+	+	+
*Drosophila biarmipes*	+	+	+	-	+	+	+
*Drosophila suzukii*	+	+	+	+	-	+	-
*Drosophila ananassae*	+	+	+	+	+	-	+
*Drosophila bipectinata*	+	+	+	+	-	+	-
*Drosophila eugracilis*	+	+	+	+	+	+	+
*Drosophila elegans*	+	+	+	+	+	+	+
*Drosophila kikkawai*	+	+	+	-	+	+	+
*Drosophila takahashii*	+	+	+	-	+	+	+
*Drosophila rhopaloa*	+	+	+	+	+	+	+
*Drosophila ficusphila*	+	+	+	-	+	+	+
*Drosophila pseudoobscura*	+	+	-	-	+	+	+
*Drosophila persimilis*	+	+	-	-	+	+	+
*Drosophila miranda*	+	+	-	+	+	+	+
*Drosophila willistoni*	+	-	+	-	-	+	+
*Drosophila virilis*	+	+	+	-	+	+	+
*Drosophila mojavensis*	+	-	-	-	+	+	+
*Drosophila albomicans*	-	-	-	-	+	+	+
*Drosophila grimshawi*	+	+	-	-	+	-	-
*Musca domestica*	+	+	-	+	-	+	-
*Anopheles gambiae*	-	-	-	-	-	-	-
*Apis mellifera*	-	-	-	-	-	-	-
*Tribolium castaneum*	-	-	-	-	-	-	-
*Aedes aegypti*	-	-	+	-	-	-	-
*Aedes albopictus*	-	-	-	-	-	-	+
*Anoplophora glabripennis*	-	+	-	-	-	-	-
*Bactrocera dorsalis*	-	-	-	+	+	-	+
*Bactrocera latifrons*	+	-	-	+	-	+	-
*Bactrocera oleae*	-	-	-	+	+	-	-
*Ceratitis capitata*	-	-	-	-	+	+	+
*Dendroctonus ponderosae*	-	+	-	-	-	-	-
*Lucilia cuprina*	-	-	+	+	-	-	+
*Plutella xylostella*	-	+	-	-	-	-	-
*Rhagoletis zephyria*	-	+	-	-	+	+	-
*Stomoxys calcitrans*	+	-	-	-	-	+	-
*Zeugodacus cucurbitae*	-	-	-	-	+	+	-

Motifs identified in the seven Ftz target genes (*en*, *drm*, *Sema5c*, *Ppa*, *Cad74A*, *noc* and opa) in each of the species marked by +, served for the generation of the motif logo using the WebLogo application (weblogo.berkeley.edu).

### Multiple Ftz target genes contain functionally important Bridge and DPE core promoter motifs

**Fig 5:**
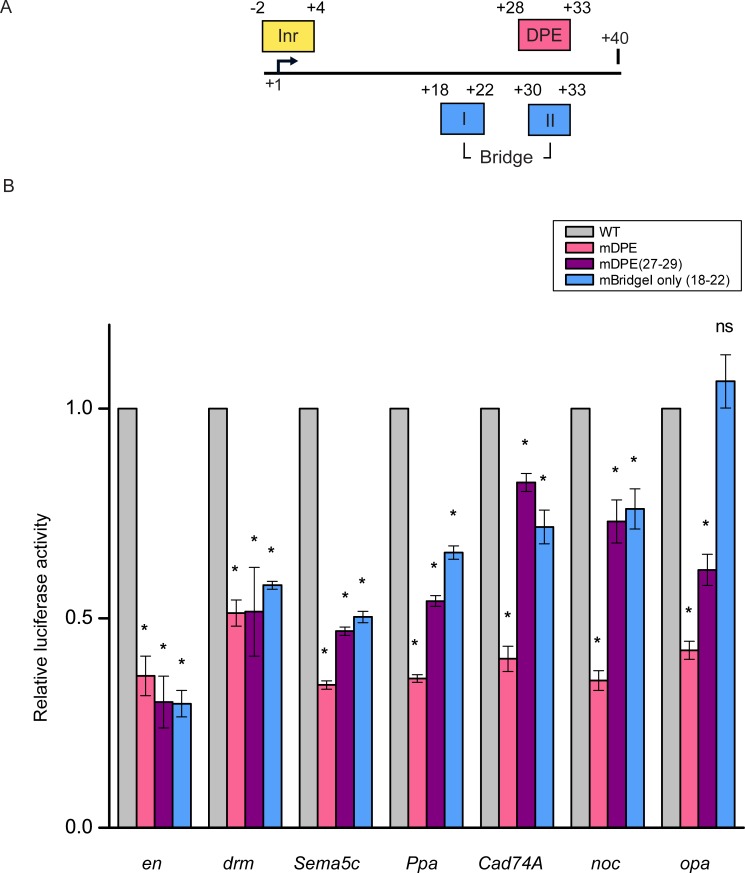
Ftz target genes are dependent on the DPE motif. (A) A schematic illustration of the downstream most common core promoter elements found in focused *Drosophila melanogaster* promoters. (B) *Drosophila* S2R+ cells were transfected with luciferase reporters that are driven by either WT, mDPE, mDPE only (27–29) or mBridgeI only (18–22) versions of the minimal promoters of *en*, *drm*, *Sema5c*, *Ppa*, *Cad74A*, *noc* and *opa*. To control for variations in transfection efficiencies, cells were co-transfected with the *Scr-Renilla* luciferase reporter and assayed for dual luciferase activities. The graph depicts the luciferase activities relative to the activities of the WT reporter plasmid, which were defined to be 1. N = 3 and error bars represent the S.E.M. Asterisks represent statistical significance, as compared to the mock version.

To examine whether the detected highly conserved DPE and Bridge sequence motifs are functional, we have generated reporter plasmids containing different versions of the minimal promoters (-10 to +40 relative to the A_+1_) for the seven Ftz target genes analyzed above. In order to distinguish between the effect of the DPE and/or the Bridge motif on the reporter activity, we have generated four distinct core promoter versions for each gene—WT, mutant DPE (mDPE), mDPE only (27–29) and mBridgeI (18–22) (Table 1). The location of these motifs is depicted in Fig 5A.

Mutations in the DPE sequence of each of the seven target genes reduced the activities of the luciferase reporter by more than 2-fold (Fig 5B). Partial mutations of the relevant regions (mDPE 27–29 and mBridgeI) had milder effects on the activities of *Cad74A* and *noc* luciferase reporter genes, as compared to their effects on the activities of the luciferase reporters driven by *en*, *drm*, *Sema5c and Ppa*. In addition, the effect of mBridgeI was more pronounced for *en*, *drm*, *Sema5c* and *Ppa*, and less pronounced for *Cad74A* and *noc* reporter genes. As expected, the mBridgeI had no effect on the activity of *opa*, which lacks a BridgeI motif. Taken together, these results emphasize the contribution of the downstream core promoter nucleotide composition to the transcriptional activity.

**Fig 6:**
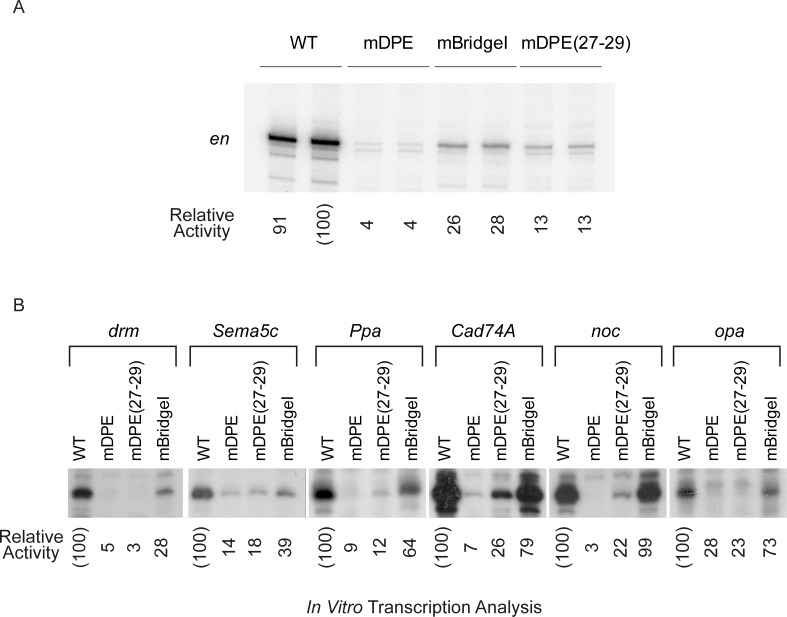
Ftz target genes are dependent on the DPE motif. (A) WT, mDPE, mBridgeI only (18–22), and mDPE only (mD27-29) version *of en*, as well as (B) WT, mDPE, mDPE only (mD27-29) and mBridgeI only (18–22) versions *of drm*, *Sema5c*, *Ppa*, *Cad74A*, *noc*, *and opa* core promoters (from –10 to +40 relative to the A_+1_ start site) were subjected to *in vitro* transcription analysis with a *Drosophila* embryo nuclear extract. The resulting transcripts were subjected to primer extension analysis using a reverse luciferase primer. Quantitation of reverse transcription products was carried out using ImageJ. Experiments testing the *en* series (A) were carried out in duplicates a minimum of two independent times to ensure reproducibility of the data. Experiments testing the *drm*, *Sema5c*, *Ppa*, *Cad74A*, *noc* and *opa* (B) were carried out a minimum of three independent times to ensure reproducibility of the data.

To gain a better understanding of the functionality of the DPE and BridgeI motifs, we analyzed the core promoter constructs by *in vitro* transcription using *Drosophila* embryo nuclear extracts, followed by primer extension analysis (Fig 6). This enables the examination of the transcriptional output of the promoters using transcription factors present in the *in-vivo* environment of the developing *Drosophila* embryo. Consistent with the luciferase reporter activities in S2R+ cells, reduced transcription levels were also observed for the mDPE versions of these genes by *in vitro* transcription and primer extension analysis, suggesting that the transcription of these Ftz target genes is DPE-dependent in both cells and embryos. Interestingly, *opa* showed the least reduction upon DPE mutation. In addition, whereas transcription of the mBridgeI versions of *en*, *drm*, *Sema5c* and *Ppa* were markedly reduced compared to WT, transcription of the mBridgeI version of *noc* was unaffected and transcription of *Cad74A* and *opa* was only moderately reduced.

The luciferase assay quantitates the enzymatic activity of a reporter gene as an indirect measure of promoter activity, whereas the primer extension assay analyzes the levels of cDNA generated from transcribed mRNA as a measure of promoter activity. Although there are some expected differences between the two assays and although the differences may result from the absence of certain transcription factors in the S2R+ cells, the overall conclusion of these combined results is that transcription of the examined Ftz target genes is significantly impaired upon mutation of the DPE motif, and that the BridgeI motif contributes to the expression of most of these Ftz target genes.

## Discussion

### *Drosophila melanogaster* Ftz and Caudal homeodomain transcription factors share functional characteristics

The *Drosophila* homeodomain transcription factor *ftz* evolved from an ancestral homeotic gene to obtain a novel function in segmentation [61]. Caudal is also a homeodomain transcription factor. Anecdotally, it is believed that *caudal* genes have a common function in axis elongation and segmentation in diverse short-germ arthropods and that this function of *caudal* most probably represents an ancestral function, deriving from the common ancestor of all arthropods [62, 63]. Here we discovered another common characteristic of Caudal and Ftz (together with its co-factor), *i*.*e*., their ability to preferentially activate gene expression via the DPE core promoter motif. We have previously shown that *Drosophila melanogaster* Caudal, as well as the mouse Caudal-related homeobox (Cdx) proteins (mCdx1, mCdx2, and mCdx4), preferentially activate transcription of the *ftz* promoters via the DPE core promoter motif [22, 57], and we now demonstrate the preference of Ftz and its co-factor Ftz-F1 for activating transcription from a DPE- versus TATA-dependent core promoter. Firefly luciferase activity values from plasmids containing the natural Ftz/Ftz-F1 binding site could not be normalized for transfection efficiency due to normalization bias, despite the use of multiple promoters driving the *Renilla* luciferase as normalization controls. This was likely due to the fact that genomic *ftz* reporters receive input from multiple cellular regulators. Thus, we have used a unique experimental setting utilizing reporter plasmids containing synthetic Ftz and Ftz-F1 binding sites. It remains to be determined whether CBP, which has been shown to contribute to DPE-preferential activation [57], may also be involved in Ftz-regulated transcription.

### The Inr, Bridge and DPE motifs contribute to the promoter activity of multiple Ftz target genes

We observed an enrichment of genes that contain a combination of Inr, Bridge and DPE motifs in Ftz target genes, as compared to the core promoter composition of the *Drosophila* genome. We thoroughly analyzed the promoter regions of seven *Drosophila melanogaster* Ftz target genes: the well-characterized *en* [32, 35, 38], the *drm*, *Sema5c*, and *noc* Ftz target genes that were identified in Ftz-F1 mutants [36] and the less characterized *Ppa*, *Cad74A* and *opa* genes [47, 60]. We demonstrated that the downstream core promoters of multiple Ftz target genes (*en*, *drm*, *Sema5c*, *Ppa*, *cad74A* and *noc*) contain functional Inr, BridgeI and DPE motifs. Interestingly, this further highlights the similarity between the well characterized and the less characterized Ftz target genes.

As demonstrated by both reporter assays in cell culture and *in vitro* transcription analysis using embryo extracts, the transcriptional output is mostly dependent on the DPE motif, with different downstream regions, including the BridgeI motif, contributing to it. This is in line with a sequence bias revealed in position +19 in DPE promoters [20].

The DPE motif was previously shown to be important for the regulation of the Hox genes, as well as for the dorsal-ventral gene regulatory network [22, 64]. Here we demonstrate its regulatory importance to the transcriptional output of several Ftz target genes, which might contribute another layer to transcriptional regulation of the segmentation gene network.

In general, these results further highlight the importance of the downstream core promoter region for transcriptional regulation of Ftz target genes, among other developmental programs. We demonstrated that BridgeI is an “auxiliary” element, which supports the function of DPE-dependent transcription in the examined promoters, but is not sufficient for fully restoring the transcriptional activity upon DPE loss. This is also supported by the fact that most examined functional DPE motifs are accompanied by the Bridge motif, while non-functional ones usually lack it. Potentially, this could promote robustness, where a strong DPE accompanied by a Bridge motif ensures proper docking of TFIID for accurate transcriptional activity.

### The downstream core promoter combination of Inr, Bridge and DPE within the promoters of Ftz target genes is mostly conserved in *Diptera*

The conservation of the combination of Inr, BridgeI and DPE motifs, as well as the strict spacing between them, may suggest the functionality of these regulatory sequences in other *Drosophila* species. Interestingly, based on this analysis, most of the identified motifs in non-*Drosophiladea* belong to the *Diptera* order. In fact, the only gene with orthologs outside *Diptera* is *drm*, which is highly conserved and has homologs in orders that include beetles (Coleoptera), butterflies and moths (Lepidoptera). Although the Ftz-F1 co-factor is conserved to bilaterians, no non-Dipteran Ftz orthologs have been identified (Flybase.org). This may explain why the specific core promoter combination is conserved to *Diptera* and additional insects, but not to higher multicellular organisms. This is another indication that it is Ftz, rather than Ftz-F1, that has the ability to activate with a preference for the specific core promoter motif. This adds another layer of complexity to this complex developmental transcriptional network. Taken together, we identified functional evolutionarily conserved downstream core promoter elements that are important for the transcriptional regulation of Ftz target genes.

## Supporting information

S1 Fig Suggested similarity between Ftz and Caudal proteins. Ftz and Caudal protein sequences, with Homeobox domain highlighted in yellow. Additional similarities, as detected by BLASTP, are indicated. Each unique color indicates a different stretch of similarity. Graphical representation of the similarity stretches was generated using MyDomains—Image Creator (Prosite, https://prosite.expasy.org/cgi-bin/prosite/mydomains/).
(PDF)

S2 Fig Ftz targets examined in this study are co-expressed with *ftz* and *ftz-f1* in the developing *Drosophila melanogaster* embryo. Expression levels of *ftz*, *ftz-f1* (orange color tones) and the examined Ftz targets (cyan color tones) were based on modENCODE data [59], as presented in FlyBase.
(PDF)
