# Supplementary material for: Identification of evolutionarily conserved downstream core promoter elements required for the transcriptional regulation of Fushi tarazu target genes
Source: PLoS One. 2019 Apr 18;14(4):e0215695. doi: 10.1371/journal.pone.0215695 (PMC6472829; doi:10.1371/journal.pone.0215695)
Supplement: S1 Fig — Ftz and Caudal protein sequences, with Homeobox domain highlighted in yellow. Additional similarities, as detected by BLASTP, are indicated. Each unique color indicates a different stretch of similarity. Graphical representation of the similarity stretches was generated using MyDomains—Image Creator (Prosite, https://prosite.expasy.org/cgi-bin/prosite/mydomains/). (PDF) [file pone.0215695.s001.pdf]

Ftz (410 aa)

```
MATTNSQSHYSYADNMNMYNMYHPHSLPPTYYDNSGSNAYYQNTSNYQGYYPQESYSESCYYYNNQ
EQVTTQTVPPVQPTTPPPKATKRKAEDDAASIIAAVEERPSTLRALLTNPVKKLKYTPDYFYTTVE
QVKKAPAVSTKVTASPAPSYDQEYVTVPTPSASEDVDYLDVYSPQSQTQKLKNGDFATPPPTTPTS
LPPLEGISTPPQSPGEKSSSAVSQEINHRIVTAPNGAGDFNWSHIEETLASDCKDSKRTRQTYTRY
QTLELEKEFHFNRYITRRRRIDIANALSLSERQIKIWFQNRRMKSKKDRTLDSSPEHCGAGYTAML
PPLEATSTATTGAPSVPVPMYHHHQTTAAYPAYSHSHSHGYGLLNDYPQQQTHQQYDAYPQQYQHQ
CSYQQHPQDLYHLS
```

Cad (427 aa)

```
MVSHYYNTLPYTQKHSAANLAYASAAGQPWNWTPNYHHTPPNHQFLGDVDSSHAAHHAAAAHQMYY
NSHHMFHSAAAASAGEWHSPASSTADNFVQNVPTSAHQLMQQHHHHAHASSSSASSGSSSSGGAP
GAPQLNETNSSIGVGGAGGGGGVGGATDGGPGSAPPNHQQHIAEGLPSPPITVSGSEISSPGAPTS
ASSPHHHLAHHLSAVANNNNNNNNNNNSPSTHNNNNNNNSVSNNNRTSPSKPPYFDWMKKPAYPAQ
PQPGKTRTKDKYRVVYTDFQRLELEKEYCTSRYITIRRKSELAQTLSLSERQVKIWFQNRRAKERK
QNKKGSDPNVMGVGVQHADYSQLLDAKAKLEPGLHLSHSLAHSMNPMAAMNIPAMRLHPHLAAHSH
SLAAVAAHSHQLQQQHSAQMSAAAAVGTLSM
```

HOMEOBOX

BlastP similarity

```
Ftz  258  RQTYTRYQTLELEKEFHFNRYITRRRRIDIANALSLSERQIKIWFQNRRMKSKKDRTLDS  317
          R  YT +Q LELEKE+  +RYIT RR+ ++A  LSLSERQ+KIWFQNRR K +K     S
Cad  277  RVVYTDFQRLELEKEYCTSRYITIRRKSELAQTLSLSERQVKIWFQNRRAKERKQNKKGS  336

Ftz  318  SPEHCGAG  325
           P   G G
Cad  337  DPNVMGVG  344


Ftz  342  GAPSVPVPMYHH  353
          G P    P YHH
Cad  27   GQPWNWTPNYHH  38
```

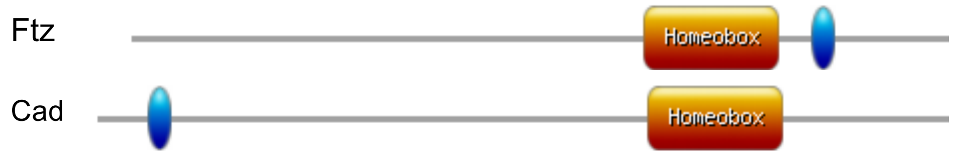

S1 Fig. Suggested similarity between Ftz and Caudal proteins. Ftz and Caudal protein sequences, with Homeobox domain highlighted in yellow. Additional similarities, as detected by blastP, are indicated. Each unique color indicates a different stretch of similarity. Graphical representation of the similarity stretches was generated using MyDomains - Image Creator (Prosite, https://prosite.expasy.org/cgi-bin/prosite/mydomains/).
