# Supplementary material for: Identification of evolutionarily conserved downstream core promoter elements required for the transcriptional regulation of Fushi tarazu target genes
Source: PLoS One. 2019 Apr 18;14(4):e0215695. doi: 10.1371/journal.pone.0215695 (PMC6472829; doi:10.1371/journal.pone.0215695)
Supplement: S2 Fig — Expression levels of ftz, ftz-f1 (orange color tones) and the examined Ftz targets (cyan color tones) were based on modENCODE data [59], as presented in FlyBase. (PDF) [file pone.0215695.s002.pdf]

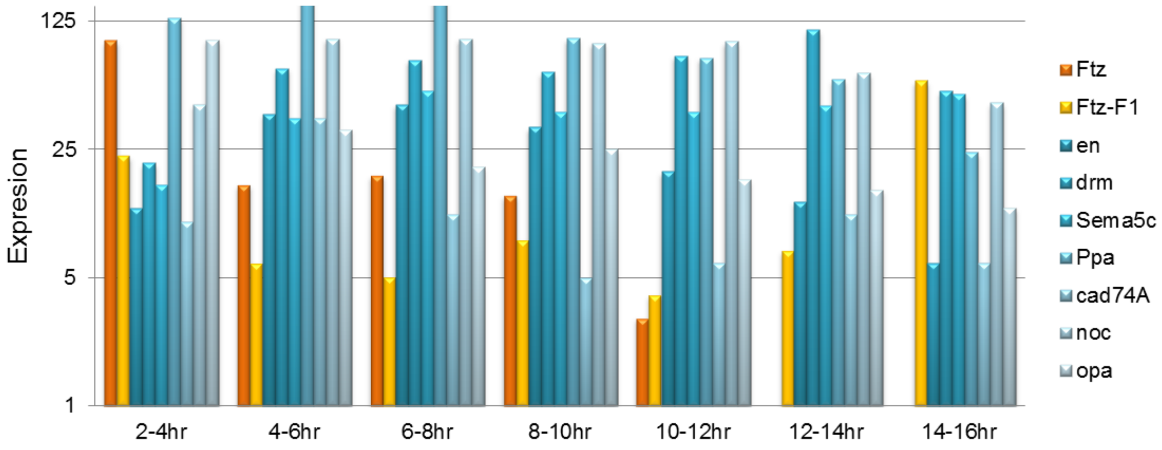

S2 Fig. Ftz targets examined in this study are co-expressed with *ftz* and *ftz-f1* in the developing *Drosophila melanogaster* embryo. Expression levels of *ftz*, *ftz-f1* (orange color tones) and the examined Ftz targets (cyan color tones) were based on modENCODE data [59], as presented in FlyBase.
